# COVID-19 Effects on the Relationship between Cryptocurrencies: Can It Be Contagion? Insights from Econophysics Approaches

**DOI:** 10.3390/e25010098

**Published:** 2023-01-03

**Authors:** Dora Almeida, Andreia Dionísio, Isabel Vieira, Paulo Ferreira

**Affiliations:** 1 CEFAGE, IIFA—Center for Advanced Studies in Management and Economics, University of Évora, Largo dos Colegiais 2, 7004-516 Évora, Portugal; 2 VALORIZA—Research Center for Endogenous Resource Valorization, 7300-555 Portalegre, Portugal; 3 Department of Economic Sciences and Organizations, Polytechnic Institute of Portalegre, 7300-555 Portalegre, Portugal

**Keywords:** contagion, cryptocurrencies, integration, mutual information, transfer entropy

## Abstract

Cryptocurrencies are relatively new and innovative financial assets. They are a topic of interest to investors and academics due to their distinctive features. Whether financial or not, extraordinary events are one of the biggest challenges facing financial markets. The onset of the COVID-19 pandemic crisis, considered by some authors a “black swan”, is one of these events. In this study, we assess integration and contagion in the cryptocurrency market in the COVID-19 pandemic context, using two entropy-based measures: mutual information and transfer entropy. Both methodologies reveal that cryptocurrencies exhibit mixed levels of integration before and after the onset of the pandemic. Cryptocurrencies displaying higher integration before the event experienced a decline in such link after the world became aware of the first cases of pneumonia in Wuhan city. In what concerns contagion, mutual information provided evidence of its presence solely for the Huobi Token, and the transfer entropy analysis pointed out Tether and Huobi Token as its main source. As both analyses indicate no contagion from the pandemic turmoil to these financial assets, cryptocurrencies may be good investment options in case of real global shocks, such as the one provoked by the COVID-19 outbreak.

## 1. Introduction

Cryptocurrencies are relatively new and innovative financial assets. They are a topic of interest to investors and academics due to their distinctive features (decentralization, blockchain technology), high returns, and apparent independence from conventional assets [1], but also for their frequent association with speculative bubbles, potential financial instability, and contagion risk [2]. The increasing number of cryptocurrencies, their levels of market capitalization, negotiation volumes, and prices [3] justify the development of analyses aimed at improving knowledge about their behaviour and co-movements, especially in periods of crisis.

Globalization promoted interdependence between financial markets and institutions [4] and has enhanced the probability of financial contagion. The literature on contagion is large and varied in methodologies and crisis contexts (see, for example, [5]). However, despite the many published studies on the subject, there is no consensus about its definition (see [6,7]). In [8], five definitions that rely on distinct assessment methodologies are presented. In fact, [9] points out that there is a coherence between the definition of contagion and the methodological approach chosen to detect it.

In this study, we adopt one of the broadest definitions, proposed by [6]. According to these authors, contagion is “a significant increase in cross-market linkages after a shock to one country (or group of countries).” [6] (p. 2223). Based on this definition, in what follows, we consider that contagion is related to a significant increase in correlation levels between cryptocurrencies, provoked by the onset of the COVID-19 pandemic. If no significant increase in correlations is detected following the cut-off moment (crisis), then we conclude that there is no contagion, although there may be interdependence.

The literature addressing issues related to the cryptocurrency market is in quick development. Several studies have evaluated the interdependence between cryptocurrencies [10] and their co-movements [11], cryptocurrency markets’ information flows [12], and links with other financial assets or markets [13,14]. Previous analyses have also assessed herding behaviour [15], co-explosivity [16], and contagion in cryptocurrency markets [17]. Our analysis uses two econophysics techniques, mutual information (MI) and transfer entropy (TE), both theoretic information-based approaches, to assess integration and contagion.

MI detects statistical dependence, regardless of the specific characteristics of the data, and captures the structure of statistical correlation [18], allowing it to be interpreted as a measure that quantifies the information exchanged between two systems. However, as a symmetric measure, it provides no evidence of the dynamics or the direction of the information exchange [19], which are both useful in evaluations of financial contagion. Thus, TE is also used to identify the direction of the information flow between pairs of cryptocurrencies and to increase the robustness of the MI analysis.

The COVID-19 pandemic has had a major effect on the global economy [20]. It has also impacted financial markets and was the first major worldwide real shock since the first cryptocurrency (Bitcoin—BTC) was launched in 2009. Natural disasters and pandemics are a source of contagion in global financial markets and an emerging line of research (see [21,22]). Financial contagion may be the outcome of both financial and non-financial crises, but in either case, assessments require the definition of a cut-off moment. This analysis is focused on the onset of the COVID-19 pandemic. Thus, in line with several studies (see, for instance, [23,24,25,26,27]), we chose 31 December 2019, as the cut-off moment, which was the day when the World Health Organization was first notified of the pneumonia cases detected in Wuhan. This was the date when information became public and available to all and, thus, to investors.

Our main goal is to evaluate integration and contagion effects in the cryptocurrency market in the COVID-19 pandemic context, specifically on its onset. We use a set of 16 cryptocurrencies (twice the number considered by [27]), rather than the more common three or four with the highest market capitalization, where the BTC is always included (see for example [28,29,30,31,32]). Furthermore, we evaluate the relationships between all possible pairs, not only between each cryptocurrency and BTC, in search of more in-depth knowledge of the cryptocurrency market’s complex dynamics.

We contribute to the existing literature in three ways. Firstly, by considering the periods before and after the COVID-19 pandemic onset, we produce new insights about the cryptocurrency market’s behaviour when the financial system was much disturbed by an extreme external event. Secondly, we provide evidence of integration between cryptocurrencies emanating from a real, rather than a financial, crisis. Finally, we quantify the information exchanged and identify the direction of the information flows within the cryptocurrency market by applying theoretic information-based approaches, still relatively uncommon in studies focusing on the cryptocurrency market.

The remainder of the paper is organized as follows: after this introduction, Section 2 reviews the relevant literature and presents recent empirical evidence of contagion in the cryptocurrency market. Section 3 presents the data and methodology. Results are shown and discussed in Section 4, and Section 5 concludes.

## 2. Brief Literature Review

Financial deregulation and liberalization, as well as technological progress, have promoted financial integration [33]. Market integration is an important feature of international finance, given, for example, its implications for diversification, risk management, and regulatory formulation. Rising correlations across markets are relevant signs of increasing market integration (see, inter alia, [34]). Therefore, evaluations of how individual financial markets relate to each other are of interest, because integration may have positive real effects (e.g., improving economic growth and welfare), but also negative ones (e.g., augmented risk of contagion). As cryptocurrencies are financial assets included in investors’ portfolios, the analysis of the co-movements between them, and also with other markets or assets, is particularly relevant. Furthermore, the cryptocurrency market offers what other markets lack, allowing observation of its structural self-organizational process since its inception.

In this study, we are interested in the co-movements between cryptocurrencies, and this interest guides our review of the relevant literature. Most empirical studies have been developed using samples containing a relatively small number of cryptocurrencies and considering the BTC as a benchmark. This was the case of the analysis by [17], which covered the period from July 2016 to May 2019 and assessed contagion from the BTC to the other cryptocurrencies, using detrended cross-correlation analysis (DCCA). They found out that there were signs of contagion for all but one cryptocurrency, the USDT. Using copula functions, ref. [35] obtained similar results, and [36] used coherence and cross-wavelet transform techniques to assess co-movement between the BTC and five major cryptocurrencies. The results indicated that there were co-movements in the time-frequency space, with leading relationships of the BTC with Dash, Monero (XMR), and Ripple (XRP), a lagged relationship with Ethereum (ETH), and out-of-phase movements with Litecoin (LTC). In addition, ref. [37] considered the interdependence of cryptocurrencies across time and frequencies to investigate the dynamics of multiscale interdependence in a sample of five leading and liquid cryptocurrencies from 2016 to 2018. They found high levels of dependence at daily frequency scales, with the cross-wavelet transforms indicating that contagion originated from XRP and ETH.

The return/volatility spillovers between cryptocurrencies were also evaluated. While [38] measured interdependence in a sample of 18 cryptocurrencies, ref. [39] examined static and dynamic volatility connectedness amongst 8 cryptocurrencies. The former concluded that the BTC was the dominant contributor to the return and volatility spillovers, but such an outcome was not supported by the latter—their results showed tight and time-varying volatility spillovers, but the BTC was not the leading influencer. Evidence of shared leadership was obtained, for instance, by [40], which evaluated connectedness via returns and volatility spillovers across six cryptocurrencies, and concluded that there was shared leadership between the BTC and LTC, with ETH as the main net receiver, highlighting the relevance of these cryptocurrencies as linkage with many others, as was found by [41]. They also distinguished positive and negative returns, finding evidence of larger negative spillovers than positive ones (contradicting [42]). XRP and ETH were the main receivers of negative-return shocks, while very weak positive-return spillovers were reported for ETH and Dash. Using intraday data of the most popular 12 cryptocurrencies, ref. [41] found that there are relevant return and volatility spillovers between them. However, when they analysed the hierarchical clustering of their sample, return- and volatility-clustering structures were quite different from each other, suggesting that return and volatility might have different spillover patterns. Analysis by [43] exposed a market with frequent structural breaks, which tended to spread from the smaller to the larger cryptocurrencies. Similarly, ref. [44] found evidence of spillover leadership by small cryptocurrencies. This diversity of results justifies the interest in further assessments.

The COVID-19 pandemic has disturbed stock markets in several countries, a fact that justifies the interest in assessments of its contagion effects in other financial markets, and never has the designation of contagion been more appropriate. In fact, despite its real nature and absence of financial roots (in contrast to, for example, the 2007/2008 subprime crisis in the US or the 2010/2011 Euro area sovereign debt crisis), the COVID-19 pandemic provoked financial turmoil [45], intensified uncertainty, caused investor panic [46], and prompted significant falls in several markets. This was the first global disturbance in the short life of cryptocurrencies [47]. Real and financial markets suffered its consequences [48,49], and cryptocurrencies are probably not an exception [50].

The impact of the COVID-19 pandemic on the cryptocurrency market has been assessed in several studies (e.g., [51,52,53,54], among others). Different timescales have also been used, with high-frequency data (such as 5 min, 10 min, 15 min, 20 min, 30 min, and 1 h, among others) specially used to analyse the network structure of cryptocurrencies and to identify cryptocurrency communities (see, for example, [54]) or to test the statistical relationship between the cryptocurrency trading volume and returns (see, for example, [55]). A broad set of these studies has used different indicators of the severity of the COVID-19 pandemic, financial-market-based proxies of down-market times, and proxies of market volatility (including cryptocurrency-market-specific volatility indices), to analyse safe-haven and hedge properties of cryptocurrencies (see, for example, [56] for a brief literature review on this issue), with mixed evidence about these properties.

In order to assess the impact of the pandemic on the cryptocurrency market, and considering a network perspective, ref. [57] estimated the multivariate transfer entropy for a set of 146 cryptocurrencies. The turmoil has changed the structure of the cryptocurrency network (in line with [51,52], among others) and led to an intensification of information flows between cryptocurrencies, coincident with the abrupt fall in stock exchanges across the world. This could indicate an increase in systematic risk and warn for the possibility of contagion. Using high-frequency data (hourly closing prices) for the period from January 2019 to December 2020, ref. [51] analysed the co-movements and correlations between Bitcoin and 31 of the most-tradable crypto assets. They identified significant changes in patterns of co-movements and correlations during the pandemic period. The evidence found suggests that during the COVID-19 crisis period, altcoins became more influential in comparison to pre-pandemic times. They also found that despite the influential role of Bitcoin in the digital asset ecosystem, due to recent developments in the blockchain ecosystem, crypto assets that can be categorised as dApps and protocols have become more attractive to investors than pure cryptocurrencies.

The relationships among the volatilities of five cryptocurrencies, three American indices, and the prices of oil and gold were analysed by [58]. They concluded that there were higher volatility spillovers between cryptocurrencies and lower volatility spillovers between cryptocurrencies and other financial assets. During stable periods, cryptocurrencies displayed low dynamic conditional correlations with financial assets. However, this pattern changed in early 2020. The conditional correlation among cryptocurrencies, stock indices, and oil increased, leading the authors to confirm the effect of the coronavirus contagion on such markets. Using the wavelet coherence approach and Markov’s switching autoregressive model, ref. [28] observed financial contagion between cryptocurrencies and stock markets during the COVID-19 pandemic. Other authors (e.g., [24,29,30]) also found evidence that the BTC does not act as a hedge during serious financial turmoil (such as the COVID-19 period). On the contrary, it amplifies contagion. In contrast, ref. [31] found evidence that BTC is a safe haven investment.

The generalized detrended cross-correlation coefficient was applied by [59] to analyse the impact of COVID-19 on multiscale cross-correlations among the cryptocurrency market (represented by BTC and ETH) and 20 conventional assets (currencies, stock market indices, and commodities). The authors found no significant cross-correlations in 2018 and 2019 between cryptocurrency and the other markets. However, this changed in 2020. They identified four specific periods of statistically significant cross-correlations, all of them related to the COVID-19 pandemic. In January 2020, with the drop of the S&P 500 and other US stock markets, BTC fulfilled its planned role as a hedge for risky assets, and ETH did not display significant cross-correlation with any other assets. In March 2020, both cryptocurrencies became risky assets, with positive significant cross-correlation identified between them and all the analysed markets (except for BTH vis-à-vis Swiss-franc (CHF), Euro (EUR), and Japanese yen (JPY)). With the beginning of the second COVID-19 wave, both cryptocurrencies revealed strong positive cross-correlation with traditional assets (except with the JPY). Curiously, at the end of August 2020, after the COVID-19 slowdown in the United States, both cryptocurrencies became positively correlated with all traditional assets, including the JPY. These findings of cross-correlations during the sharp market falls in early 2020, and also during the recovery phase, may be a sign that cryptocurrencies became more connected with other global financial markets.

Using the same approach, but extending the analysed period and including four more stock indices, ref. [59,60] concluded that the cryptocurrency market was strongly cross-correlated during the turbulent periods of the COVID-19 pandemic, but it showed even higher levels of cross-correlations with the other markets during the same turbulent periods. However, even in such periods, the cryptocurrency market is more independent from the other markets than those markets are independent among themselves, and the cross-correlations between the cryptocurrency market and the other ones tend to decrease as the pandemic becomes a more normal component of everyday life. Aiming to evaluate the connectedness among 27 emerging equity markets and seven high-capitalized cryptocurrencies before and during the COVID-19 pandemic, ref. [53] applied the network connectedness approach of Diebold and Yılmaz (2014). They found that the correlations within and between the cryptocurrency and equity markets strengthened after the coronavirus outbreak. While the crypto market assets were mostly risk transmitters before the pandemic, this risk-distributing role shifted more to the equity markets after the pandemic outbreak. The analysis of frequency connectedness shows that major cryptocurrencies cannot be used as diversifiers for the emerging stock markets because of their very high eigenvector centrality scores.

The return spillovers between 18 cryptocurrencies in low and high volatility regimes were evaluated by [47] between July 2016 and April 2020. They found several spillovers in both regimes. A rolling windows analysis produced evidence of significant structural changes in spillovers, not only in late 2018, but also in early 2020. Much higher spillovers in the high volatility regime were observed during the COVID-19 outbreak, which is consistent with the existence of contagion. Covering a short period, from January 2020 to April 2021, ref. [61] examined the impact of COVID-19 on the connectedness of eight cryptocurrencies. The results showed, firstly, that cryptocurrencies act as a net receiver and transmitter of shocks (the BTC and ETH were the highest transmitters). Secondly, the causality-in-quantile test showed that the pandemic significantly caused spillover connectedness between cryptocurrencies. Extending the analysed period until 31 October 2020, ref. [60] investigated the presence of detrended cross-correlations on the 80 most liquid cryptocurrencies listed on Binance. Applying a spectral analysis of the detrended correlation matrix and topological analysis of the minimal spanning, they concluded (in line with [62]) that the cryptocurrencies became more strongly cross-correlated among themselves than they used to be, and that the average cross-correlations increased with time on a specific time scale (similar to the Epps effect). They also found changes in the topology of the minimal spanning trees, which became more centralized for short time scales and more distributed (and also more correlated) for long time scales.

The cryptocurrency market, like other financial markets, is dynamic. Its properties are therefore constantly changing and evolving, and are still far from being fully identified and understood [60], maybe because most past research was focused exclusively on BTC, or at most on the four or five most important cryptocurrencies [63].

Most studies of contagion, interdependence, or integration in this market consider samples of the main cryptocurrencies (for example the study of [64], which analysed the multifractal cross-correlations of BTC and ETH trading characteristics in the post-COVID-19 time) and evaluate the relationship between each one and the BTC. Other possible links that are, nevertheless, potentially important to improve knowledge of the dynamics of this complex market have been less explored. Furthermore, except for [27], those studies are focused on the connectedness across cryptocurrencies, while we assess the information shared in the cryptocurrency market, using mutual information and the transfer entropy concepts (both based on information-theoretic measures). In order to fill such gaps, this study considers a sample of 16 cryptocurrencies and evaluates relationships between all possible pairs.

Of interest for analyses of contagion is the fact that the COVID-19 pandemic presents distinct features from past crises. Potential contagion from the COVID-19 outbreak has one clear catalyst that can be timestamped. This differentiates the pandemic crisis from the other well-researched economic and financial sources of contagion, for which there were various probable turmoil catalysts. In such cases, it could be difficult to exactly pinpoint what provoked the crisis, which creates noise in contagion assessments. For example, in the case of the 2007–2008 subprime crisis, there were various underlying causes.

Most analyses evaluate contagion when its source is of a financial nature. The trading volume of cryptocurrencies has broken records during the COVID-19 pandemic. This adds to the importance of examining their interactions, market dynamics, and the return spillovers in a set of cryptocurrencies that are representative of this market. We perform such assessments using distinct, but complementary methods, which are described and justified in the next section.

## 3. Data and Methods

The empirical analysis is developed considering 31 December 2019, as the cut-off date separating the pre-crisis and the COVID-19 crisis periods. The data sample comprises closing daily prices for 16 cryptocurrencies (twice the number used by [27]) with more than a billion-dollar market capitalization on 7 March 2020, representing more than 94% of the total market capitalization on that date (the total market capitalization of all the cryptocurrencies available in the used database was 263,364,575,633 USD on 7 March 2020, the moment of data retrieval). The sample is, thus, representative of the cryptocurrency market. Furthermore, the less well-known and less capitalized a cryptocurrency is, the less liquid and less reliable the related data is [60], thus justifying the use of cryptocurrencies with higher levels of market capitalization. The data were obtained from an open-access source (https://coinmarketcap.com, accessed on 31 January 2021), considered an appropriate database to conduct research [65]. The sample was selected in order to cover various degrees of market capitalization and different underlying business models for cryptocurrencies. Due to data availability constraints, the time series for the distinct cryptocurrencies have different starting dates. All data available before the cut-off moment were considered in order to preserve all the possible information contained in each time series. All series end by 30 January 2021 (see Table 1 for more details). Daily returns for the cryptocurrencies are calculated as $r_{i,t}=\ln\left(\frac{P_{i,t}}{P_{i,\;t-1}}\right)$, where $r_{i,t}$ is the return of cryptocurrency *i* at period *t*, and $P_{i,t}\;$ and $P_{i,t-1}$ are the prices at time *t* and *t* − 1, respectively.

The objective is to evaluate the cryptocurrencies’ co-movements before and during the COVID-19 pandemic. This allows us to conclude in terms of increased integration, contagion, or independence, in line with our adopted definition of contagion (produced, as mentioned above, by [6]), but also with those of authors such as [8] (definitions 3 and 4) or [34].

As the returns do not follow a multivariate normal distribution, linear approaches might not be the most suitable. In contagion and in integration assessments, more than identifying cross-correlations, it is important to find the information flows and sources. Thus, since theoretic information-based approaches (which are econophysics approaches) allow this, MI and TE are both applied.

The application of physics concepts to understand economic phenomena is the basis of econophysics. Econophysics is a branch of literature that results from the connection between statistical physics and economics. The concept of entropy is the central concept of statistical mechanics, which is the main branch of physics that underlies econophysics. Econophysics approaches have been used for more than a quarter century to study financial markets, and they have allowed a better understanding of the markets’ complex behaviour and of the mechanisms governing various phenomena in these markets (e.g., speculative bubble formation, market crashes, asset cross-correlations, nonlinear autocorrelations, the efficacy of investing strategies, price formation, etc.). It has been applied in many areas of economics, including financial markets’ dynamics. Some of these applications are ontological and others are metaphorical, as they draw on models of information theory (due to [66]) or other models using the mathematics of entropy theory [67]. It is common to resort to information theory, and especially to the concept of entropy, to express numerically an amount of information that is shared or transferred between various data sets.

MI is a symmetric and bivariate measure of independence. It is based on the concepts of entropy and divergence, proposed respectively by [66,68]. It can aggregate the concepts of uncertainty and information. It may be applied to infer the existence of dependence. According to the latter authors, it is a measure of the difference between two probability distributions. Considering two discrete random variables, X and Y, with marginal probability distributions, $p_{X}\left(x\right)$ and $p_{Y}\left(y\right)$, respectively, and joint probability distribution $p_{X,Y}\left(x,y\right)$, MI is given by:

(1) $$I\left(X,Y\right)=\sum_{x,y}p_{X,Y}\left(x,y\right)\times log\frac{p_{X,Y}\left(x,y\right)}{p_{X}\left(x\right)p_{Y}\left(y\right)}$$

The MI between two processes is then a measure of the reduction of uncertainty (or information gain) in relation to a state where the two processes are independent ($p_{X,Y}\left(x,y\right)=p_{X}\left(x\right)p_{Y}\left(y\right)$). In case of independence, the MI is null. One of our goals is to assess contagion in the cryptocurrency market, and this can be done by comparing the MI before and after the onset of the pandemic.

According to [44], TE is an alternative to traditional causality assessments. It allows estimation of the information flow between series, without requiring the pre-specification of a model, nor a specific data structure (linearity), and has outperformed in quantifications of the bi-directional flow of information [69]. TE is also robust to spurious relations [70]. To quantify the information flow in a financial context, specific time-series properties and an asymmetric measure are required [71]. A dynamic (considering transition probabilities instead of static ones) and directional structure (by adding time lags to the variables) was introduced by [19]. Aiming to measure the information flow between two series, [19] joined the Shannon entropy and the Kullback and Leibler distance concepts, considering that stationary Markov processes are involved. In the bivariate case, the information flow from Y (an l order process) to X (a k order process) can be measured by quantification of the deviation to the generalized Markov properties. This implies that the probability of observing  X  in time t+1  in the state x conditional to the k  previous observations is given by $p\left(x_{t+1}|x_{t}^{\left(k\right)}\right)=p\left(x_{t+1}|x_{t}^{\left(k\right)},y_{t}^{\left(l\right)}\right)$, with $x_{t}^{\left(k\right)}=(x_{t},\ldots ,\;x_{t-k+1}$) and $\;y_{t}^{\left(l\right)}=(y_{t},\ldots ,\;y_{t-l+1}$), allowing the definition of TE, provided in Equation (2). According to [19], the Shannon TE capturing the information flow from Y to X is given by:

(2) $$TE_{Y\to X}\left(k,l\right)=\sum_{x_{t+1},x_{t}^{\left(k\right)},y_{t}^{\left(l\right)}}p\left(x_{t+1},x_{t}^{\left(k\right)},y_{t}^{\left(l\right)}\right)log\frac{p\left(x_{t+1}|x_{t}^{\left(k\right)},y_{t}^{\left(l\right)}\right)}{p\left(x_{t+1}|x_{t}^{\left(k\right)}\right)}$$

If $p\left(x_{t+1}|x_{t}^{\left(k\right)}\right)=p\left(x_{t+1}|x_{t}^{\left(k\right)},y_{t}^{\left(l\right)}\right)$, then TE=0,  and the state of Y does not influence the transition probabilities of X.

In a “gaussian world”, Granger Causality (GC) and TE are coincident [72], but this is not the case in nonlinear dynamic contexts, and nonlinearity is common in financial markets’ data (and, thus, probably in the cryptocurrency market, as well). Therefore, TE could be a better measure to analyse the dynamics and possible asymmetric information flows between cryptocurrencies. GC is a causal information flow measure (from a source to a destination), while TE is a directional and dynamic measure of predictive information. The cryptocurrency market is a complex system, and the cryptocurrencies display complex, probably nonlinear, behaviour. Thus, the use of TE is justified for an in-depth evaluation of their relations, including their magnitudes. All series of returns are stationarity, and thus, the TE can be used.

TE, defined in Equation (2), is directional, as it considers dependence originating in *Y*. The degree of dependence from X relatively to Y shows its asymmetry. $TE_{X\to Y}\left(k,l\right)$ can be calculated following an identical procedure. The information flow’s dominant direction may be computed by subtracting TEs [19]. If $TE_{Y\to X}\left(k,l\right)-TE_{X\to Y}\left(k,l\right)>0,\;$ the dominant direction of the information flow is from Y  to X; if $TE_{Y\to X}\left(k,l\right)-TE_{X\to Y}\left(k,l\right)<0,\;$ the opposite occurs (the dominant direction of the information flow is from X  to Y). If $TE_{Y\to X}\left(k,l\right)-TE_{X\to Y}\left(k,l\right)=0,\;$ the flows are equivalent in terms of dominance.

The TE measure is derived for discrete data. However, most economic time series, such as the return series, are considered continuous (not in what concerns time, but because they can take any value on a continuous scale, the real line, meaning they can also assume any fractional value). Thus, data discretization is necessary and may be done using a finite number of partitions and symbolic codification. In the case of asset returns, observations located in the tails are particularly important, and so data partition based on empirical quantiles is usual. This means that observations in the left and right tails fall into different categories. The results depend on the number of bins, and three bins are usually considered [73]. The series of returns are split into three bins across the 5% and 95% quantiles (represented by $q_{\left[0.05\right]}^{r}$ e $q_{\left[0.95\right]}^{r}$, respectively). This division is consensual in the literature (see also [7]). The symbolic coding will replace each of the values in the series under analysis, (𝑡), by the corresponding symbol, i.e.,:

(3) $$S\left(t\right)=\{\begin{array}{c} 1\;\\ 2\;\\ 3\; \end{array}\begin{array}{c} for\;y\left(t\right)\leq q_{1}\\ for\;q_{1}<y\left(t\right)<q_{2}\\ for\;y\left(t\right)\geq q_{2} \end{array}$$

The statistical significance of TE and consequent statistical inference are based on the bootstrap method proposed by [74], with 300 bootstrap replications (nboot = 300, default value) and considering 50 observations from the beginning of the bootstrapped Markov chain (burn = 50, default value). Repetition of the TE estimation produces the estimations’ distribution under the null hypothesis of absence of information flow. The *p*-value is given by $1-{\hat{q}}_{T}$, with ${\hat{q}}_{T}\;$ as the simulated distribution quantile, determined by the TE estimation [75]. TE estimations are computed using software R, R Transfer Entropy.

## 4. Results and Discussion

### 4.1. Descriptive Statistics

The descriptive statistics of cryptocurrencies’ returns are presented in Table 2. Using StataSE 15^®^ (64-bit) software, a standard Augmented Dickey–Fuller test for stationarity was also performed (to save space, these results are not shown, but are available upon request). All the series of returns are stationary ($H_{0}$ of the Augmented Dickey–Fuller test was rejected).

The results suggest that the onset of the COVID-19 pandemic did not significantly change the cryptocurrencies’ behaviour, as their volatility did not increase after 31 December 2019. On the contrary, volatility seems to have decreased. However, this should be considered with care because the number of observations is not constant across periods. The average return for most cryptocurrencies is positive and near zero. Average returns have increased since the 31 December 2019, except for Bitcoin SV (BSV) and Binance Coin (BNB).

Average returns on BSV were higher before 31 December 2019. After this date, it was the cryptocurrency with the lowest average returns and the highest volatility. Skewness was positive in the first period (with the exceptions of BTC and USDT) and negative in the second (with the exceptions of BSV, USDT, and Stellar (XLM)), corroborating [27,51]. This could be a sign of increased sensitivity to the effects of the COVID-19 pandemic and may be interpreted as a higher probability of large positive return variations than negative ones in the first period. In contrast, negative returns could be more frequent in the second period, which could reflect the turmoil and uncertainty in these markets. High kurtosis values are observed in both periods, i.e., leptokurtic distributions. This means that the returns do not follow a normal distribution. They display fat-tails (a stylized fact in financial markets), justifying the application of nonlinear, rather than linear, techniques. In the period before 31 December 2019, USDT revealed an extremely high kurtosis value. This is a stable cryptocurrency, pegged to the USD. However, shortly after it was first issued in 2014, questions of whether USDT’s issuer was really setting aside enough in assets to keep its dollar peg secure have been raised. This was in part due to the fact that the company had never released audited financial statements that normal deposit-taking banks are required to report. Only in 2017, and due to investors’ doubts, did the company start issuing attestations on its reserves. This may be a possible explanation for the high kurtosis value found in this cryptocurrency in the period before 31 December 2019. In June 2018, a report from Freeh Sporkin & Sullivan, LLP, based on a random date balance inspection and a full review of relevant documentation of bank accounts, confirmed that, as of that date, all tethers in circulation were indeed fully backed by USD reserves, which may explain the more similar kurtosis values between the USDT and the remaining cryptocurrencies in the period after the 31 December 2019.

### 4.2. Mutual Information

**Figure 1:**
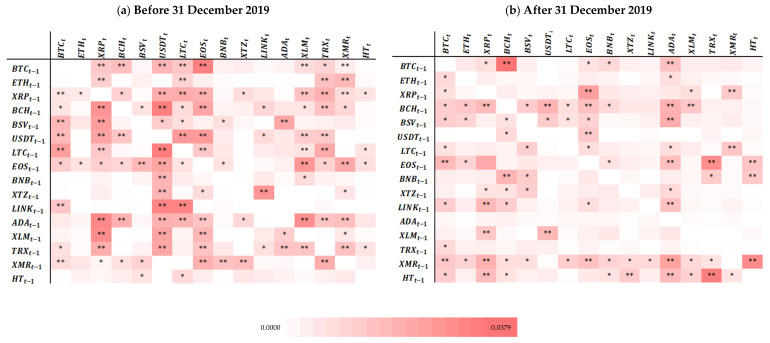
Mutual Information Heatmaps. Notes: i. Each heatmap represents the relationship between the cryptocurrencies before (**a**) and after (**b**) 31 December 2019, estimated by mutual information (to be read as the mutual information between cryptocurrencies in rows and columns); ii. A one-day lag was considered for the mutual information calculus; iii. Lighter red means lower mutual information values, while darker red means higher mutual information values. The minimum mutual information value was 0.000, and the maximum was 0.0379; iv. In order to be visually clearer, the change in mutual information between the periods use the same colour scale; v. “**” and “*” refers to the statistical significance of the relationship, with 1% and 5% significance, respectively.

Figure 1a,b display the relationship between cryptocurrency returns before and after 31 December 2019, estimated by MI and presented in a heatmap format (the values are not displayed, but are available upon request) with the significance levels, which allows for a quick analysis of the intensity and the statistical significance of the relationships. MI measures the common information between variables displayed in rows and in columns. The row variables are for moment t−1, and those in columns are for moment t. For example, $MI\left(BTC_{t-1},\;All\;the\;other\;cryptocurrencies_{t)}\right)$ and $MI\left(All\;the\;other\;cryptocurrencies_{t-1},BTC_{t)}\right)$ are estimated. This means that the diagonally opposite MI values do not have to be equal.

Although the higher colour intensity indicates more common information, i.e., higher dependence between cryptocurrencies, it does not necessarily mean that such dependence is statistically significant. The statistical significance is evaluated according to the critical values provided by [76]. Evidence of statistically significant global dependence indicates a strong relationship between cryptocurrencies, which means integration. Statistically significant MI between assets could mean a violation of the Efficient Market Hypothesis (EMH) because investors can use historical information to obtain abnormal profits. However, based on this analysis, it is not possible to assess whether abnormal profits are systematic, and, thus, nothing can be concluded about a possible violation of the EMH.

Figure 1a shows that USDT, EOS, LTC, XRP, and BTC are the least independent cryptocurrencies (displaying higher integration). ETH, BSV, BNB, Tezos (XTZ), Chainlink (LINK), Cardano (ADA), and HT are the most independent cryptocurrencies (the less integrated). As these cryptocurrencies have different levels of market capitalization and are less dependent, market capitalization is not a decisive factor in what concerns (in)dependence. The two major cryptocurrencies, BTC and ETH, are independent from each other, which could be related to their different mining protocols and different times required to mine blocks. While the BTC protocol sequences transaction into groups called blocks, ETH focuses on providing a platform to facilitate decentralized building applications on its blockchain [77]. Cryptocurrencies with higher market capitalization display lower independence, and BTC shares the leadership on return spillovers with other cryptocurrencies, not being the leader, corroborating [39,40,51,52,60], among others. Between the least and the most independent cryptocurrencies, are older and newer cryptocurrencies, thus the return series length is not a decisive factor in what concerns (in)dependence.

In order to evaluate and identify whether cryptocurrencies have become more or less integrated due to the onset of the COVID-19 pandemic, which can be interpreted as a contagion risk in these markets, we have to ascertain whether dependence between them has increased after the cut-off date. Thus, MI was also estimated after 31 December 2019, and the results are also presented as a heatmap (Figure 1b)).

After the cut-off date, the cryptocurrencies sharing the greatest amount of information with the others are BCH, BSV, EOS, XMR, and HT. BTC reduced the information shared with other cryptocurrencies, which means that there was a reduction in its level of integration. BCH was the most dependent on BTC, indicating greater integration between the two. LINK, Litecoin (LTC), ETH, BNB, and XTZ were the most independent (i.e., the less integrated) cryptocurrencies. Figure 1b suggests that, overall, the cryptocurrencies continued to be integrated. This reveals the continuity of the cryptocurrencies’ dependence structure, in line with [78]. Nevertheless, the number of statistically significant relationships decreased, leading to the conclusion that cryptocurrencies have lower dependence after the beginning of the pandemic crisis, i.e., have become less integrated, contradicting [27,51,60].

We also aimed to evaluate contagion between cryptocurrencies during the onset of the COVID-19 pandemic. To such end, we analysed the cryptocurrencies that were not integrated before the 31 December 2019 (which displayed no statistically significant relationships). If after this date, they became integrated, displaying statistically significant MI, then contagion exists. Following this rationale, there seems to be contagion between the pairs signalled in black in Table 3.

Although most cryptocurrencies exhibit signs of contagion from HT, there is no contagion from the majority of them to the others (except to ADA). We conclude that, overall, there is no contagion in our sample of cryptocurrencies.

### 4.3. Transfer Entropy Results

**Figure 2:**
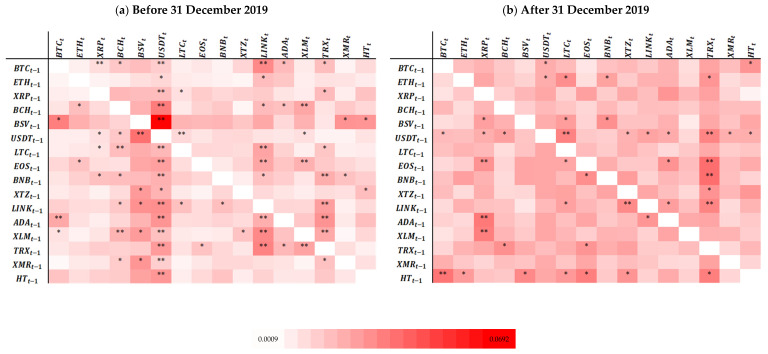
Transfer Entropy Heatmaps. Notes: i. Each heatmap represents the relationship between the cryptocurrencies analysed, before (**a**) and after (**b**) 31 December 2019, estimated by transfer entropy (to be read as the cryptocurrencies in rows influencing the cryptocurrencies in columns); ii. A one-day lag was considered for the transfer entropy calculus; iii. Lighter red means lower transfer entropy values, while darker red means higher transfer entropy values. The minimum mutual information value was 0.0021, and the maximum was 0.0692; iv. In order to be visually clearer, the change in the transfer entropy between the periods use the same colour scale; v. “**” and “*” refers to the statistical significance of the relationship, with 1% and 5% significance, respectively.

We also computed TE to identify the direction of the information flows, and as a complement to the MI analysis. Figure 2a,b show the relationship between cryptocurrencies in rows and in columns, estimated by TE (the TE values are available upon request). These heatmaps are interpreted as those displaying the MI analysis’ results, and as shown above, cryptocurrencies in rows influence the cryptocurrencies in columns.

Figure 2a shows that all cryptocurrencies (regardless of market capitalization or the dimension of their series of returns) strongly and significantly influence USDT. However, the latter only significantly influences BSV, LTC, XRP, BCH, and XLM. On the contrary, BSV significantly influences BTC, XMR, HT, and USDT, i.e., it influences the cryptocurrency with the highest market capitalization (BTC), and also the two with the lowest market capitalization (XMR and HT). BTC, EOS, ADA, TRX, LTC, and XLM have the most significant influence on all the others. Evidence concerning the last two cryptocurrencies corroborates findings by [47]. LINK, XLM, TRX, and USDT (all of them non-mineable [55]) are the most significantly influenced by the other cryptocurrencies. The first three have lower market capitalization, while the last one displays one of the highest market capitalizations. Thus, amongst the main influencers and the most influenced, we find cryptocurrencies with different levels of market capitalization, indicating that such a feature does not determine which cryptocurrencies are influencers or influenced. Between the most influential and most influenced cryptocurrencies are older and newer cryptocurrencies, suggesting that the length of the returns series is not a decisive factor in what concerns the dynamic pattern of influence.

BTC, although significantly influencing some cryptocurrencies (e.g., XRP, USDT, and LINK), is not dominant in terms of TE, corroborating [39,40,51,79], but contradicting [38,61]. This may reflect the fact that the increasing number of cryptocurrencies is responsible for a less prominent role of BTC and for an increased level of competitiveness in the cryptocurrency market.

The absence of statistical significance between cryptocurrencies indicates the absence of integration amongst cryptocurrencies. In fact, the heatmap shows the absence of statistically significant flows between most pairs of cryptocurrencies (especially if the pair contains either ETH or HT).

Figure 2b clearly shows a reduction of significant relationships between cryptocurrencies (lower dependence), and thus lower levels of integration. Amongst the cryptocurrencies more significantly influenced by the others are TRX, XRP, and LTC (again, cryptocurrencies with very different levels of market capitalization). There are significant information flows from USDT and HT to most of the other cryptocurrencies, which means that these two are the leading influencers and are integrated with almost all the others.

HT, a cryptocurrency with lower market capitalization and the second most recent in the sample, was the only one exhibiting a significant information flow to other cryptocurrencies with high capitalization (BTC and ETH). This is in line with [43,44] and can lead to a more complex structure than that of the other markets, where, typically, assets with a high level of market capitalization have spillover effects on the less capitalized ones [60].

Some cryptocurrencies receive no statistically significant influence from others (this is the case of XLM, contradicting [58]) or receive it from a maximum of two (such as BTC, ETH, BCH, BSV, USDT, BNB, LINK, XMR, and HT). This indicates that there is a high level of segmentation within the sample assessed in this study and suggests that most of these cryptocurrencies may be used for diversification purposes in portfolios of cryptocurrencies (in line with conclusions of [80]).

Analysing Figure 2a,b simultaneously, we observe that the (b) part clearly displays a relatively lower number of statistically significant relationships. Therefore, cryptocurrencies exhibit a lower level of integration, in line with the evidence produced by the MI analysis and contradicting [51].

According to [81], the transfer entropy estimation could be biased when available data are limited and the expected effect is rather small due to finite sample effects, making it difficult to assess the significance of the obtained values. A bias correction is possible and used to calculate effective transfer entropy (ETE), which is given by $ETE_{Y\to X}\left(k,l\right)=TE_{Y\to X}\left(k,l\right)-TE_{Y_{shuffled}\to X}\left(k,l\right)$, where $TE_{Y_{shuffled}\to X}$ indicates the transfer entropy, using a shuffled version of the time series. The shuffling process implies randomly drawing values from the observed time series and realigning them to generate a new time series. This process destroys the time series dependencies of *Y*, as well as the statistical dependencies between *Y* and *X* ([75]). To assess the statistical significance of the transfer entropy estimates, a Markov block bootstrap process was applied, which in contrast to shuffling, preserves the dependencies within the variables *Y* and *X*, but eliminates the statistical dependencies between them. Thus, repeated estimation of transfer entropy provides the distribution of the estimates under the null hypothesis of no information flow. We also accessed (as the R Transfer Entropy package allows it) the effective transfer entropy. However, as the transfer entropy and effective transfer entropy values quantify the amount of information flow from one series to another time series [82], and given the results obtained do not lead to different interpretations from the ones presented above, and due to space constraints, the results are available upon request.

In order to evaluate contagion between cryptocurrencies during the COVID-19 pan-demic, we follow a procedure that is similar to that adopted when considering MI results. Evidence of contagion exists solely for the pairs signalled in black in Table 4.

As TE allows the identification of directional causality between two variables, for the above black-marked pairs, the source of contagion are the cryptocurrencies lagged one period (in rows). Generally, USDT and HT seem to be the main sources of contagion. However, as for most cryptocurrencies, there are no statistically significant signs of contagion; hence, we conclude that the assessed sample has not been affected by contagion from the COVID-19 pandemic.

## 5. Conclusions

The main goal of this study was to assess contagion from the COVID-19 pandemic to the cryptocurrency market. We followed [6] in considering that contagion occurs when there is a significant increase in the links between assets or markets in the aftermath of a shock. We used MI and TE to evaluate contagion in a sample of 16 cryptocurrencies in the aftermath of the shock provoked by the onset of the COVID-19 pandemic. The TE analysis complements the MI evaluation of dynamic and directional information flows and increases its robustness.

The initial, exploratory MI analysis produced evidence of mixed integration patterns, with some cryptocurrencies linked to others (EOS is one of the least independent in both periods), and some revealing a status of independence in the context of the analysed sample (LINK, ETH, BNB, and XTZ are the most independent in both periods). Although there are mixed integration patterns in both periods, after 31 December 2019, the number of statistically significant relationships declined, indicating that the COVID-19 outbreak did not contribute to enhance integration in the cryptocurrency market. However, the pandemic seems to have produced dynamic changes in the group of analysed cryptocurrencies, and this is in line with some previous studies (e.g., [51,52,57], among others), concluding that the behaviour of the cryptocurrencies changes during periods of crisis.

The TE analysis also revealed mixed integration patterns in both periods, reinforcing the results of the MI analysis, with TRX being one of the most influenced in both periods. There were no cryptocurrencies leading return spillovers in the pre-31 December 2019 period, but after this date, USDT and HT became leading influencers. This supports previous evidence indicating that BTC does not lead, and thus is not a main player in the cryptocurrency market [4,39,51]. Therefore, changes in the behaviour of USDT and HT (the influencers in the sample) require close monitoring during pandemic periods.

Both econophysics approaches indicate that just a few cryptocurrencies were affected by the pandemic crisis. MI provided evidence of contagion between the majority of the cryptocurrencies and ADA, and between HT and almost all the cryptocurrencies in the sample. TE showed that, in general, USDT and HT were the main sources of contagion in the cryptocurrency market. The analysis provides no evidence of contagion for most assessed cryptocurrencies, suggesting that these financial assets may be a good alternative investment to consider in the context of global real shocks that impact the financial markets.

Although there is generally no evidence of contagion in the group of assessed cryptocurrencies, in a few cases (for the influenced and the influencers) there may be a potential for disturbance of the cryptocurrency market’s stability. Investors may, however, use influencer cryptocurrencies as possible predictors of the return of those influenced and to obtain information to improve knowledge when deciding on portfolio composition. As the main influencers are different in both periods, some cryptocurrencies may be used as possible return predictors in stable periods, while others are more useful in times of turmoil.

Given the indicators from the descriptive statistics and the evidence from theoretic information-based approaches, we conclude that the pandemic crisis did not strengthen integration between cryptocurrencies, in contrast with [27,51,60]. The market thus offers some portfolio diversification opportunities, a conclusion that is not surprising, given the apparent disconnection of cryptocurrencies from the real economy. There could, however, have been some contagion if the market was affected by general investors’ panic or fear, which was not the case for the analysed sample.

The main limitation of this study is the smaller number of observations available after 31 December 2019. Nevertheless, given the rapid materialization of some real effects in financial markets, our analysis provides results that are useful to inform decisions during non-financial crises. Another limitation may be the different number of observations for each time series in the period before 31 December 2019. Both issues will be considered in future research devoted to the analysis of the effect of other disturbing events on this particular market.

## Figures and Tables

**Table 1:** Sample Description.

Cryptocurrency			Start Date	Market Capitalization (USD)		Observations	
						Before 31 December 2019	After 31 December 2019
1	Bitcoin	BTC	29 April 2013	162,684,945,903	61.77%	2.437	396
2	Ethereum	ETH	07 August 2015	26,164,459,704	9.93%	1.607	396
3	Ripple	XRP	04 August 2013	26,164,459,704	9.93%	2.340	396
4	Bitcoin Cash	BCH	23 July 2017	6,059,789,428	2.30%	891	396
5	Bitcoin SV	BSV	09 November 2018	4,290,029,659	1.63%	417	396
6	Tether	USDT	25 February 2015	4,643,212,805	1.76%	1.770	396
7	Litecoin	LTC	29 April 2013	3,889,681,824	1.48%	2.437	396
8	EOS	EOS	01 July 2017	3,366,250,140	1.28%	913	396
9	BinanceCoin	BNB	25 July 2017	3,138,663,736	1.19%	889	396
10	Tezos	XTZ	02 October 2017	2,103,907,641	0.80%	820	396
11	ChainLink	LINK	20 September 2017	1,520,607,569	0.58%	832	396
12	Cardano	ADA	01 October 2017	1,268,987,677	0.48%	821	396
13	Stellar	XLM	05 August 2014	1,183,231,787	0.45%	1.974	396
14	TRON	TRX	13 September 2017	1,136,886,287	0.43%	839	396
15	Monero	XMR	21 May 2014	1,143,443,765	0.43%	2.050	396
16	Huobi Token	HT	03 February 2018	1,063,188,577	0.40%	696	396
Total				249,821,746,206	94.86%		

Note: i. Table 1 shows basic information, such as the starting date, the market capitalization of each cryptocurrency (in value and percentage) on 7 March 2020, and the number of observations before and after the cut-off date of 31 December 2019 (after the cut-off date, the number of observations is the same for all the analysed cryptocurrencies); ii. The number of observations refers to closing prices, which means that the series of returns have one less observation; iii. The total market capitalization on 7 March 2020, of all the cryptocurrencies available on the used database was 263,64,575,633 USD.

**Table 2:** Cryptocurrencies’ Returns Descriptive Statistics.

Cryptocurrency	Before 31 December 2019				After 31 December 2019			
	Mean	Stdev.	Skewness	Kurtosis	Mean	Stdev.	Skewness	Kurtosis
BTC	0.0016	0.0427	−0.1527	10.7409	0.0039	0.0414	−3.4812	44.5290
ETH	0.0024	0.0714	−3.4274	74.6109	0.0060	0.0551	−2.5411	29.9171
XRP	0.0015	0.0727	2.0756	32.9133	0.0021	0.0660	−0.3960	26.4318
BCH	−0.0008	0.0794	0.6179	10.4098	0.0018	0.0603	−1.8145	24.2868
BSV	0.0008	0.0901	0.8643	19.9132	0.0015	0.0814	2.8755	46.5471
USDT	−0.0001	0.0211	−12.2749	829.3628	0.0000	0.0055	0.1522	37.9746
LTC	0.0009	0.0645	1.7163	28.5632	0.0030	0.0540	−1.5536	16.3358
EOS	0.0010	0.0827	2.2245	27.6377	0.0030	0.0545	−2.0790	22.8957
BNB	0.0055	0.0787	1.3888	15.1944	0.0003	0.0502	−3.3523	38.3843
XTZ	−0.0004	0.0751	0.1255	10.5396	0.0019	0.0634	−2.1090	24.3520
LINK	0.0027	0.0812	0.7048	7.1339	0.0065	0.0711	−1.4227	18.0953
ADA	0.0003	0.0792	2.9094	29.3140	0.0061	0.0623	−1.1089	14.6842
XLM	0.0015	0.0754	2.0089	19.6020	0.0050	0.0668	1.6195	21.9256
TRX	0.0023	0.0963	2.1343	19.3240	0.0022	0.0545	−2.2636	24.9947
XMR	0.0016	0.0703	0.6497	9.6001	0.0029	0.0509	−2.4056	26.4712
HT	0.0009	0.0518	0.6165	7.6063	0.0021	0.0431	−3.5911	49.8863

Note: Stdev represents the standard deviation.

**Table 3:** Contagion between Cryptocurrencies under a Mutual Information Analysis.

	$BTC_{t}$	$ETH_{t}$	$XRP_{t}$	$BCH_{t}$	$BSV_{t}$	$USDT_{t}$	$LTC_{t}$	$EOS_{t}$	$BNB_{t}$	$XTZ_{t}$	$LINK_{t}$	$ADA_{t}$	$XLM_{t}$	$TRX_{t}$	$XMR_{t}$	$HT_{t}$
$BTC_{t-1}$																
$ETH_{t-1}$																
$XRP_{t-1}$																
$BCH_{t-1}$																
$BSV_{t-1}$																
$USDT_{t-1}$																
$LTC_{t-1}$																
$EOS_{t-1}$																
$BNB_{t-1}$																
$XTZ_{t-1}$																
$LINK_{t-1}$																
$ADA_{t-1}$																
$XLM_{t-1}$																
$TRX_{t-1}$																
$XMR_{t-1}$																
$HT_{t-1}$																

**Table 4:** Contagion between Cryptocurrencies under a Transfer Entropy Analysis.

→	$BTC_{t}$	$ETH_{t}$	$XRP_{t}$	$BCH_{t}$	$BSV_{t}$	$USDT_{t}$	$LTC_{t}$	$EOS_{t}$	$BNB_{t}$	$XTZ_{t}$	$LINK_{t}$	$ADA_{t}$	$XLM_{t}$	$TRX_{t}$	$XMR_{t}$	$HT_{t}$
$BTC_{t-1}$																
$ETH_{t-1}$																
$XRP_{t-1}$																
$BCH_{t-1}$																
$BSV_{t-1}$																
$USDT_{t-1}$																
$LTC_{t-1}$																
$EOS_{t-1}$																
$BNB_{t-1}$																
$XTZ_{t-1}$																
$LINK_{t-1}$																
$ADA_{t-1}$																
$XLM_{t-1}$																
$TRX_{t-1}$																
$XMR_{t-1}$																
$HT_{t-1}$																

## Data Availability

The data presented in this study are available on request from the corresponding author.
